# Onset of Telomere Dysfunction and Fusions in Human Ovarian Carcinoma

**DOI:** 10.3390/cells8050414

**Published:** 2019-05-04

**Authors:** Nazmul Huda, Yan Xu, Alison M. Bates, Deborah A. Rankin, Nagarajan Kannan, David Gilley

**Affiliations:** 1 Department of Pathology and Laboratory Medicine, Indiana University School of Medicine, Indianapolis, IN 46202, USA; nhuda@iu.edu; 2 Department of Obstetrics and Gynecology, Indiana University School of Medicine, Indianapolis, IN 46202, USA; xu2@iu.edu; 3 Biochemistry and Molecular Biology, Indiana University School of Medicine, Indianapolis, IN 46202, USA; alibates@iu.edu; 4 Department of Chemistry and Applied Biological Science, South Dakota School of Mines and Technology, Rapid City, SD 57701, USA; deborah.rankin@mines.sdsmt.edu; 5 Division of Experimental Pathology, Department of Laboratory Medicine and Pathology, Mayo Clinic, Rochester, MN 55905, USA; Kannan.Nagarajan@mayo.edu

**Keywords:** telomere, telomere dysfunction, ovarian carcinoma, telomerase, genomic instability

## Abstract

Telomere dysfunction has been strongly implicated in the initiation of genomic instability and is suspected to be an early event in the carcinogenesis of human solid tumors. Recent findings have established the presence of telomere fusions in human breast and prostate malignancies; however, the onset of this genomic instability mechanism during progression of other solid cancers is not well understood. Herein, we explored telomere dynamics in patient-derived epithelial ovarian cancers (OC), a malignancy characterized by multiple distinct subtypes, extensive molecular heterogeneity, and widespread genomic instability. We discovered a high frequency of telomere fusions in ovarian tumor tissues; however, limited telomere fusions were detected in normal adjacent tissues or benign ovarian samples. In addition, we found relatively high levels of both telomerase activity and hTERT expression, along with anaphase bridges in tumor tissues, which were notably absent in adjacent normal ovarian tissues and benign lesions. These results suggest that telomere dysfunction may occur early in ovarian carcinogenesis and, importantly, that it may play a critical role in the initiation and progression of the disease. Recognizing telomere dysfunction as a pervasive feature of this heterogeneous malignancy may facilitate the future development of novel diagnostic tools and improved methods of disease monitoring and treatment.

## 1. Introduction

The telomere, terminal chromosomal tandem DNA repeats and associated proteins, prevents the cellular DNA repair machinery from recognizing chromosome ends as double strand breaks [1]. Telomerase is a special DNA polymerase with an integral RNA moiety that acts as a template for the synthesis of telomeric DNA [2]. Human mitotic somatic cells generally do not contain sufficient levels of telomerase to maintain telomere length, which causes telomere shortening with each cell cycle due to the “end replication problem” presented by conventional cellular DNA polymerases. Gradually, telomere function is lost with continual erosion of telomeric DNA, which can lead to unprotected chromosome ends and subsequent chromosome end-to-end fusions (telomere fusions) [3,4]. Cells with potentially only one or a few critically shortened telomeres are likely eliminated via senescence and/or cell death pathways [5,6,7]. However, a very rare cell (or cells) may bypass these cellular elimination pathways resulting in telomere dysfunction-induced genomic instability via breakage-fusion-bridge cycles. Additionally, continual modes of telomere dysfunction may induce genomic instability throughout tumorigenesis [8]. Almost two decades ago, it was discovered that high levels of human telomerase activity are present in the majority of human solid tumor cells. However, the exact mechanism that leads to high levels of telomerase activity in human tumor cells remains largely unknown [2].

Although the general role of telomere dysfunction in the development of human cancer has been widely acknowledged, direct evidence of damaged telomeres in human cancer has begun to appear [9]. Shortened telomeres and telomere fusions have been detected in blood samples from patients with early stage chronic lymphocytic leukemia, and telomere shortening and fusion events were found to increase as the disease advanced [10]. Research has also shown the presence of telomere fusions in early human breast carcinoma (DCIS, ductal carcinoma in situ) and invasive ductal carcinoma, while fusions in normal tissues were notably absent [11]. Additionally, benign prostatic hyperplasia (BPH), high-grade prostatic intraepithelial neoplasia (PIN), and prostate cancer (PCa) prostate lesions all contain similarly high frequencies of telomere fusions, while tumor-adjacent, histologically normal prostate tissue generally did not contain telomere fusions [12]. Taken together, this evidence suggests that telomere fusions may occur early in tumorigenesis and may function as a driving force in cancer development, genetic heterogeneity, and progression. 

Ovarian cancer is the most common cause of death from gynecologic malignancies with 10-year survival rates less than 30% [13]. Due to diagnosis at late, advanced stages, these cancers are exceedingly challenging to treat and therefore identification of early molecular changes associated with development of ovarian cancers has been an area of intense interest. Characterization efforts, however, have only rendered the disease more frustratingly complex, as detailed examinations of histological profiles and genetic aberrations have revealed a wide array of critical gene mutations, copy number alterations, variations in gene expression, and epigenetic modifications that vary dramatically between ovarian cancer subtypes and even divide some subtypes into further delineated categories [14,15]. The vast degree of heterogeneity found in ovarian cancer increases the necessity of finding common, unifying features of the disease that can be used to develop new methods of early detection. Our hypothesis is that human ovarian tumor tissue contains significant amounts of telomere fusions, as found in the other human carcinomas (breast and prostate) on which we have previously reported [11,12]. 

The high-grade serous cancer (HGSC) subtype alone account for ~90% of ovarian cancer associated deaths [16]. Patients who harbor hereditary mutations in BRCA1, BRCA2, BRIP1, PALB2, RAD51C and RAD51D have been shown to exhibit a higher risk of developing HGSC [16]. It is interesting to note the recent paradigm shift related to the cell of origin for the predominant subtype of ovarian cancer i.e., HGSC. Using a *Dicer-Pten* double-knockout mouse model, Matzuk’s team was able to trace ovarian cancers to fallopian tube mesenchyme and demonstrated that removal of fallopian tube, not ovaries, led to prevention of HGSC [17]. Most recently, Velculescue et al. provide convincing evolutionary evidence of the metastatic progression of HGSC in ovaries from precursor lesions in fallopian tube tissue using laser captured microdissected tissues and whole-exome sequence analysis [18]. These and other studies point toward a possible shared developmental origin for epithelial ovarian cancers.

In this current study, we utilized a multiplex PCR-based telomere fusion assay called TAR (telomere associated repeat) fusion PCR to examine adjacent normal, benign, and malignant ovarian human tissues. Our method allowed a quantitative analysis of the frequency of telomere fusions from human solid tissues [11,12]. The major goal of this study is to determine the extent of telomere dysfunction as reflected the telomere fusion accumulation in human ovarian tumor tissues. We also assessed indicators of telomere dysfunction such as shortened telomere lengths and anaphase bridges, which suggest onset of genomic instability and are frequently associated with telomere fusion-break-fusion cycles. In addition, we evaluated telomere length, telomerase activity, and mRNA levels of the reverse transcriptase protein subunit of the telomerase enzyme (hTERT). Telomere fusions were found in significant quantities in ovarian tumor tissues, while limited telomere fusions were detected in “normal” adjacent tissues or benign ovarian human tissue samples. Furthermore, telomerase activity was elevated in 100% of tumor tissues examined, but was not detectable in normal and benign tissues. Overall, our data demonstrates widespread telomere dysfunction in ovarian cancer and suggests that telomere dysfunction may occur at early stages of ovarian cancers. Continual cycles of telomere dysfunction likely drives additional genomic instability, which may lead to metastatic transformation in ovarian cancers and may play a pivotal role in the establishment of disease in these patients. 

## 2. Materials and Methods

### 2.1. Ovarian Tissues 

“Normal” tumor adjacent tissue, benign tumor tissue, and malignant tumor tissue (25–80 years old; mean age 55 ± 16 years) were obtained from the IU Cancer Center Tissue Bank at the IU Simon Cancer Center using an Indiana University School of Medicine Institutional Review Board-approved protocol. Informed consent was obtained in writing from all participants involved. Immediately following surgical removal, tissue specimens were placed in iced, sterile containers and transferred to Pathology for diagnosis and processing. Each portion of deidentified tissue was given a unique barcode for identification. Each tissue specimen contained approximately 100–250 mg of tissue. All tissue samples were obtained by excisional biopsy and were examined histologically by pathologists at the IU Simon Cancer Center for analysis. Additional information on tissue samples utilized in this study is provided in Table 1 and Table 2. 

### 2.2. DNA/RNA/Protein Isolation 

DNA, RNA, and protein were isolated from ovarian tissue samples using the AllPrep DNA/RNA/Protein Mini Kit (Qiagen, Hilden, Germany, Catalog #80004) according to provided protocol. RNA isolated with this protocol was used to synthesize cDNA using the QuantiTect Reverse Transcription Kit (Qiagen, Catalog #205311) according to provided protocol. Resulting DNA, RNA, and protein concentrations in each sample were assessed using a Nanodrop 2000 spectrophotometer (Thermo Fisher Scientific, Waltham, MA, USA). 

### 2.3. TAR Fusion PCR 

Two-step touchdown PCR was performed in an Applied BioSystems Veriti Thermal Cycler in a 20 μL reaction mixture using 50 ng of DNA, 8 different primers, 10 mM 7-deaza-dGTP (Roche Diagnostics), and Advantage GC Genomic LA Polymerase Mix (Clontech). PCR cycling conditions included 1 denaturation cycle of 3 minutes at 94 °C, 10 cycles each of 30 seconds at 94 °C and 5 minutes of a temperature diminishing from 72 °C to 68 °C by 0.4°C with each successive cycle (the “touchdown”), and 20 additional cycles of 30 seconds at 94 °C and 5 minutes at 68 °C. Primer Mix A contained eight primers that anneal within TAR1 regions (with the exception of Xp and 17p): 1p, 2p, 5p, 7q, 9p, 11q, 12q, 15q, 16p, 17p, 18p, Xp, and Xq. Primer Mix B contained eight primers that anneal within TAR1 regions (with the exception of Xp and 17p): 1q, 2p, 4p, 4q, 5p, 7q, 10q, 12q, 17p, 18p, 19q, 21q, and Xp. Primer sequences are listed in publication by Tanaka et al. [11]. TAR fusion PCR products were then resolved on a 0.8% agarose gel, and Southern blot analysis was performed using a ^32^P-labeled [TTAGGG]_4_ probe. Normal male DNA and BJ foreskin fibroblast cell DNA were used as negative controls. BJ cells expressing the human papillomavirus type 16 E6/E7 oncoproteins (BJ E6/E7) DNA and ductal carcinoma in situ (DCIS) breast tumor tissue DNA, both previously identified to exhibit numerous telomere fusions were used as positive controls [19]. 

To calculate the total number of possible telomere fusion combinations, the general equation *C(n,2) + n* was used, where *n* is the number of unique chromosomal ends. This equation can be simplified to *n(n+1)/2*. In human somatic cells with 46 possible unique chromosome ends, there are (46 × 47)/2 = 1081 possible end-to-end fusion combinations. Each primer mix covers 13 chromosome ends (*n* = 13), so each primer mix can detect (13 × 14)/2 = 91 possible fusion combinations. Primer Mix A and Primer Mix B share 7 primers for a total number of (7 × 8)/2 = 28 shared fusion combinations. Thus, the total number of possible fusion combinations detected by both primer mixes is 91 + 91 – 28 = 154, for a total percentage of coverage of 154/1081 = 14.2%. [11,20].

### 2.4. Telomere Length

Telomere length of ovarian tissue DNA was assessed using the Cawthon method [21]. Six 20 μL reactions per tissue sample were assayed by qPCR in a Roche LightCycler 480 under the following cycling conditions: 1 cycle of 10 minutes at 94 °C, 2 cycles of 10 seconds at 94 °C followed by 15 seconds at 49 °C, and 35 cycles of 10 seconds at 94 °C followed by 15 seconds at 62 °C followed by 30 seconds at 74 °C. Reaction mixtures with no template DNA (nuclease-free water only) were used as negative controls.

### 2.5. Telomerase Activity

Telomerase activity in ovarian tissue lysates was measured by Telomeric Repeat Amplification Protocol (TRAP) using the TRAPeze Telomerase Detection Kit (Millipore). Tissue were lysed with ice cold 1x CHAPS lysis buffer, and one 50μL reaction per tissue sample was prepared using 0.5 μg of protein extract, 10× TRAP Precision Buffer, dNTPs, primers, and 2 U of Taq DNA polymerase. Reaction mixtures were subjected to the following PCR cycling conditions in an Eppendorf MasterCycler Gradient: 1 cycle of 30 minutes at 30 °C, 1 cycle of 5 minutes at 95 °C, and 33 cycles of 30 seconds at 95 °C followed by 30 seconds at 52 °C followed by 30 seconds at 72 °C. To view telomerase-generated bands, PCR products were electrophoresed on a 12.5% polyacrylamide gel and stained with SYBR Green (Roche Diagnostics). A heat-inactivated sample was used as a negative control, and a positive control was provided with the kit.

### 2.6. hTERT Expression

Three reactions per tissue sample were assayed with the TaqMan system as described by Bièche et al. [22] using a Roche LightCycler 480 and probes from the Roche Universal Probe Library (Roche Diagnostics). Reaction mixtures with no template DNA (nuclease-free water only) and DNA from telomerase-negative BJ cells were used as negative controls. DNA from HeLa cells was used as a positive control, and hTERT expression in tissue samples was quantitated by comparison with hTERT expression in the control HeLa cells. 

### 2.7. Analysis of Anaphase Bridges

All formalin fixed-paraffin embedded tissues were received from the Indiana University Simon Cancer Center Tissue Bank with Indiana University School of Medicine Institutional Review Board approval. Tissue blocks were sectioned and stained with Haematoxylin and Eosin (H&E) and scored microscopically (Leica DN5000B) for anaphase bridges in ovarian tumor tissue and corresponding adjacent, histologically normal ovarian tissue [23,24]. An anaphase bridge was defined as a continuous chromatin link between two separated chromosome masses at anaphase [25].

## 3. Results

**Figure 1:**
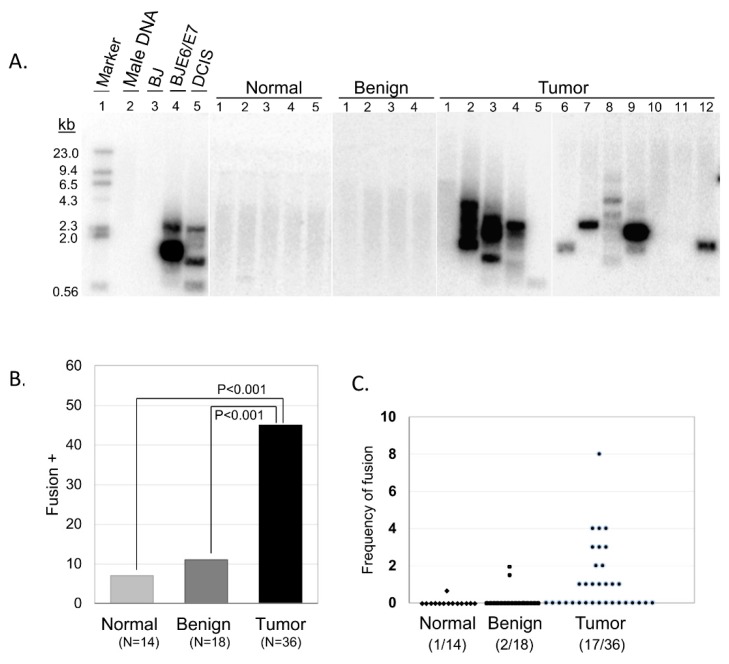
Telomere fusions in the ovarian tissue samples. (**A**) Southern blot analysis using ^32^P-labled [TTAGGG]_4_ probe. Five representative samples are shown for normal and benign, and 12 for tumor tissue samples. Normal male DNA and BJ foreskin fibroblast cells are used as negative controls, while BJ cells expressing the human papillomavirus type 16 E6/E7 oncoproteins (BJ E6/E7) DNA and ductal carcinoma in situ (DCIS) DNA are used as positive controls. (**B**) The percentage of fusion-positive samples. Standard t-test analysis is shown. (**C**) Frequency of telomere fusions where each dot represented one individual with the number of telomere fusion Southern bands per 500ng genomic DNA from five TAR-Fusion PCRs.

We used our multiplex TAR fusion PCR assay to investigate the presence of telomere end-to-end fusions in ovarian tissue specimen obtained from oophorectomy surgeries [11]. Following patient consent, we collected 54 tissues including 18 benign and 36 malignant tissues. We also collected 14 contralateral tumor-free ovarian tissues. The collection of primers used in our assay has been calculated to cover approximately 14% of all possible chromosomal end-to-end combinations (see *Materials and Methods* section for detailed calculation information) due to the currently limited availability of known telomere adjacent sequences that can be used for primer synthesis. Despite this limitation in coverage of potential chromosomal end-to-end fusions, 16 out of 36 tumor samples (44%) displayed clear fusion bands on Southern blot analysis, while adjacent normal and benign samples showed limited telomere fusions (1 out of 14 normal adjacent tissues, 7%; 2 out of 18 benign tissues, 11%) (Figure 1). These results highlight a distinct difference between non-malignant and malignant tissue, and illustrate the predominance of telomere dysfunction as a shared characteristic of ovarian malignancies.

**Figure 2:**
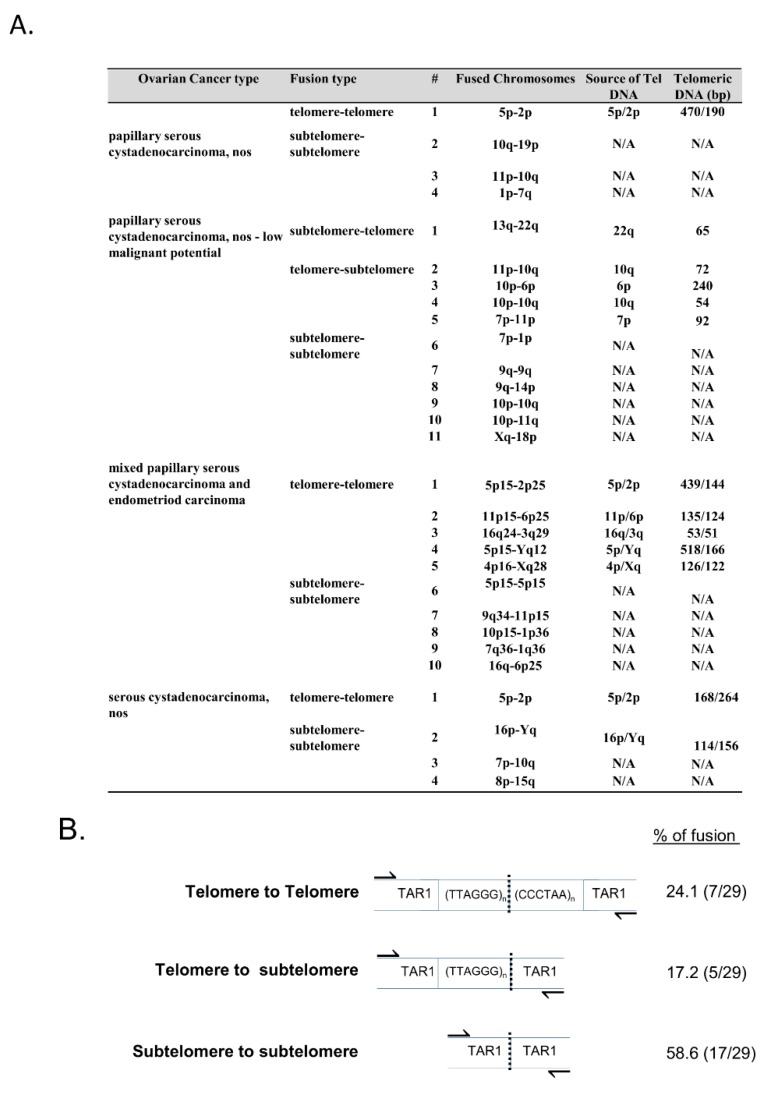
Telomere length and fusion analysis. (**A**) Each tumor tissue sample was evaluated for telomere length using qPCR. Each sample’s fusion type, categorization of fused chromosomes, and telomeric DNA (bp) length is shown. (**B**) Breakdown of the types of telomere fusion found in the above samples.

Telomere fusion junction sequence analysis revealed that the majority of telomere fusion junctions from ovarian tumor tissue contained subtelomeric DNA to subtelomeric DNA fusion junctions lacking telomeric DNA (58.6%; Figure 2A,B). Additionally, we found fusion junctions with relatively short telomeric DNA tracts from one chromosome fused to subtelomeric DNA regions from another distinct chromosome end (17.2%; Figure 2A,B), and short telomeric DNA to short telomeric DNA fusion junctions containing telomeric DNA likely from both chromosomes involved in the fusion events (24.1%; Figure 2A,B). Previously, we reported that fusion junctions within DCIS (grade 3) breast tumor tissue did not contain significant levels of microhomology at telomere fusions junctions [11]. The telomere fusion junctions examined within this ovarian tumor tissue study also revealed no significant levels of microhomology.

**Figure 3:**
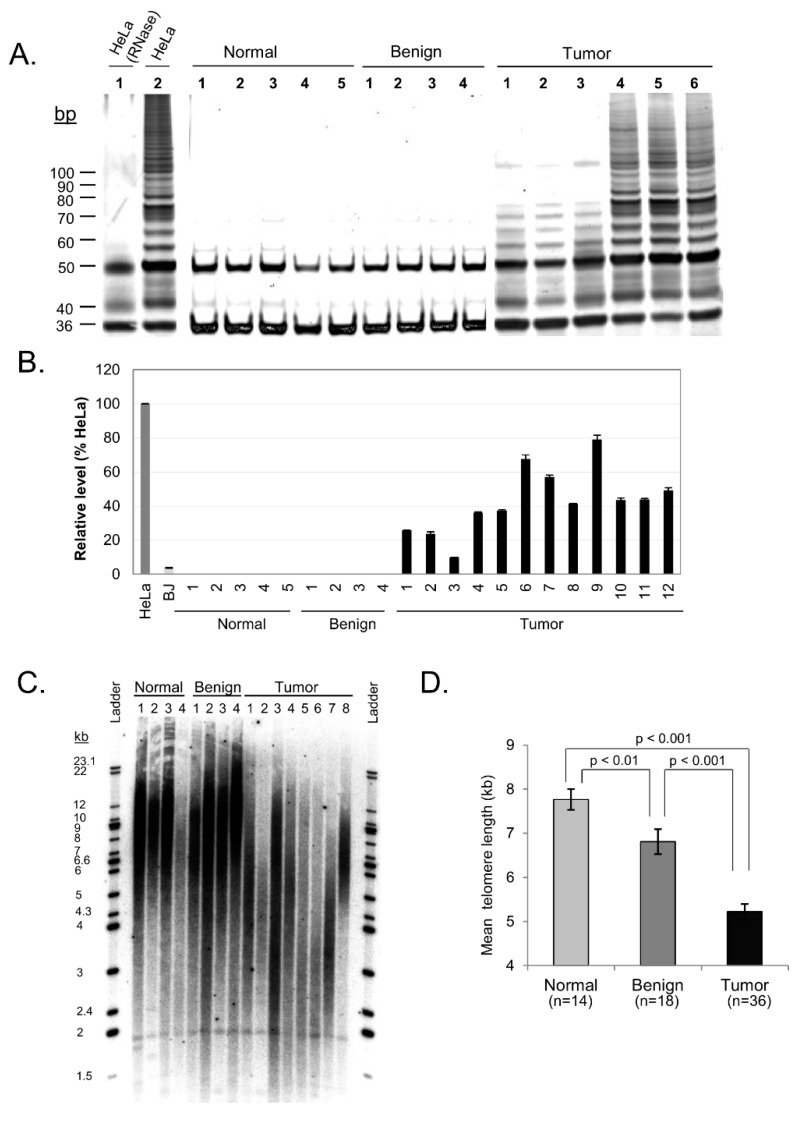
hTERT, telomerase, and telomere length profiles of ovarian carcinoma tissue samples. (**A**) Representative samples with telomerase activity in ovarian carcinoma. Fresh lysates of HeLa cells were used as positive control for telomerase activity while heat treated HeLa cells with inactivated telomerase serve as negative control. (**B**) Representative samples of hTERT expression levels in ovarian carcinoma quantified. HeLa and BJ cells were used as positive and negative controls respectively. (**C**) Representative Southern Blot of telomere length comparing normal, benign and tumor ovarian tissue. (**D**) Quantified mean telomere length (kb) of normal, benign and tumor tissue samples (mean ± SE). Standard t-test analysis is shown.

To further elucidate the dynamics of telomere maintenance in ovarian tissue, we next assessed activity of the telomerase enzyme in each tissue sample using the telomeric repeat amplification protocol (TRAP). Minimal to no appreciable telomerase activity was detected in adjacent normal and benign samples, while notable elevation in telomerase activity was observed in 100% of tumor tissue samples (Figure 3A and Table 1). Although this data was not quantitatively analyzed, it nevertheless provides qualitative empirical evidence that a clear difference exists between non-tumor and tumor ovarian tissue with regard to telomerase activity – a distinction that correlates with hTERT mRNA expression levels in these samples as described below.

mRNA expression of the aforementioned catalytic subunit of the telomerase enzyme, known as hTERT, was measured in ovarian tissue by comparison with hTERT expression in control HeLa cells. Analysis of hTERT expression in each tissue sample found that neither the adjacent normal nor the benign tissue samples contained detectable levels of hTERT mRNA, while 19 of the 20 tumor samples displayed substantial hTERT expression (P = 0.035 between adjacent normal and tumor tissue; Figure 3B). Tumor tissue samples on average exhibited 49% of the hTERT expression of HeLa cells. These results correlate strongly with the qualitative telomerase activity data obtained via the TRAP assay. This suggests that the telomerase enzyme is actively synthesized in malignant ovarian tissue and may play a significant role in telomere maintenance and cancer progression in these malignancies.

Since degradation of the telomere has been implicated in the initiation of telomere fusions and subsequent breakage-fusion-bridge cycles, we also examined telomere length in our samples. Telomere length was determined by qPCR and showed significant differences between tissue categories (Figure 3C,D). Mean telomere length for adjacent normal ovarian tissue was 7.85 kb (SE = 0.40), benign tissue showed shorter telomeres at a mean length of 6.65 kb (SE = 0.12), and ovarian tumor tissue samples showed even shorter telomeres at a mean length of 5.2 kb (SE = 0.15). Our results suggest that progressive shortening of telomere length may contribute to telomere dysfunction in ovarian carcinoma.

**Figure 4:**
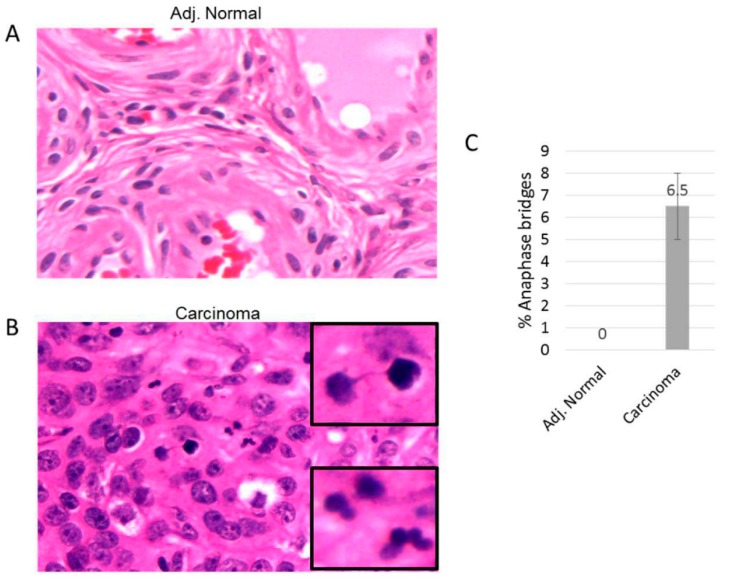
Anaphase bridge analysis in ovarian carcinoma. Tissue sections are stained with Hematoxylin and Eosin (H&E). (**A**) and (**B**) are representative adjacent tissue and ovarian carcinoma tissue sections, respectively. (**C**) The percent of anaphase bridges was calculated from the total anapases within each ovarian carcinoma.

We performed anaphase bridge analysis for ovarian tumor lesions to determine if additional markers of genomic instability associated with telomere dysfunction were specifically detected in ovarian lesion epithelial lesions as opposed to other tissue cell types. We examined sectioned tissues from ovarian tumor lesions along with corresponding adjacent normal tissue from each lesion from 10 patients (Figure 4). We found that epithelial cells, but not other cell types, from ovarian tumor lesions contained significant levels of anaphase bridges (Figure 4B). Corresponding normal adjacent tissue from each ovarian lesion did not contain detectable levels of anaphase bridges (Figure 4A). These results highlight the presence of additional marker for genomic instability (anaphase bridges) within ovarian tumor tissue and suggest that epithelial cells may be the cell type of origin of telomere fusions.

## 4. Discussion

We determined the extent of telomere dysfunction in human epithelial ovarian carcinoma in order to evaluate whether this form of genomic instability plays a role in ovarian carcinogenesis. Specifically, we searched for evidence of telomere fusions, which are known to initiate breakage-fusion-bridge cycles that can subsequently lead to extensive genomic damage and instability, a prime suspect in the development of certain cancer types [26]. Our findings support the hypothesis that telomere fusions are a prevalent event during ovarian malignancies, as 44% of the tumor tissue samples (given the limitation of current assay as previously discussed in the results section and below) assayed were positive for telomere fusions, whereas adjacent normal and benign tissues showed significant lower levels of telomere fusion. Our data is consistent with the genomic evolution model of solid tumors that is also applicable to ovarian cancer [27]. Based on our results and the evolution model, we speculate that the relatively preserved genomic integrity in benign tissue is the characteristic of the majority of prevalent subclones in these tissue samples, but a rare, albeit undetectable tumor subclone(s) with compromised telomere integrity and aneuploidy, may expand in number and become increasingly detectable during the malignant growth phase. As mentioned previously, the current limitations of our assay enable us to detect only 14% of the possible chromosome end-to-end telomere fusion combinations. In spite of this limitation, 44% of the tumor tissues assayed exhibited the presence of telomere fusions. Such a high prevalence of telomere fusions, derived from such a limited number of possible associations with the current assay indicates that telomere fusions are frequent and widespread events in ovarian carcinoma. Furthermore, this strongly suggests that a substantial fraction of the 56% of tissues that did *not* exhibit detectable levels of telomere fusions would likely be positive for telomere fusions if the remaining 86% of possible end-to-end chromosome fusion combinations could be detected in the telomere fusion assay with increased coverage. Although it is possible that certain chromosome ends may fuse more frequently than others, our previous study of breast cancer [11], along with this current report, in which fusion junctions were sequenced, showed no particular prevalence of any specific types of fusion between specific chromosomes. In addition, since the primer combinations utilized in this assay were determined randomly, solely by virtue of unique terminal chromosomal DNA sequences at the time of assay development, it is unlikely that certain telomere ends are more likely to form fusions in the current 14% coverage range. Therefore, in light of the apparently stochastic nature of fusions indicated by our previous work, it is improbable that fusions in ovarian cancer would occur at significantly different frequencies. This data provides striking evidence for the pervasive and potentially ubiquitous nature of telomere dysfunction in ovarian cancer, a unifying characteristic of a profoundly heterogeneous disease that may act as a driving force in tumorigenesis.

Previously published data on ovarian cancer has shown that ovarian tumors exhibit significantly shorter telomeres than normal tissues [28,29,30]. Other research, however, has indicated that changes in telomere length can include both shortening and elongation and that telomere length has no significant relationship to the stage of disease. [31,32]. One difficulty in assessing the importance of telomere length is the fact that assays performed on frozen tissue samples only provide a snapshot of telomere dynamics at one specific moment in time. Investigators are left to discern whether or not the conditions observed in telomere maintenance have reached a stable, static state or if they are moving toward one extreme or the other. Captured in a brief moment in time, it is difficult to know if the telomere lengths measured in an assay represent a constant, stable length that has remained at that level for an indefinite period of time, if they are in the process of shortening with successive cell divisions, or if telomere-elongation pathways such as activated telomerase or alternative lengthening of telomeres (ALT) [33] may be lengthening them and rescuing them from genomic crisis. Each individual tumor may differ from others in this regard, and intra-tumoral heterogeneity may even cause differences in telomere dynamics within the same tumor. Malignant cells in a tumor tissue may feature critically short telomeres, while non-malignant neighboring cells may have telomeres of a more normal length. This potentially broad variety of telomere length in the cells of a single tumor may skew the mean telomere length measurements for these tissues and prevent an accurate comparison of tissue types. Given these potential complications with determining telomere length in tumor tissue samples, here we found significant shorting of telomere lengths in ovarian tumor tissue cells compared to adjacent normal ovarian tissue cells and benign ovarian tissue cells. Therefore, progressive shortening of telomere length appears to likely contribute to telomere dysfunction in ovarian carcinoma.

hTERT expression and telomerase activity illuminate yet another facet of ovarian cancer telomere dynamics. While the RNA component of the telomerase enzyme (known as hTR) is expressed constitutively in both adjacent normal and malignant tissues [34,35], expression of the catalytic subunit hTERT is highly regulated in human cells. Believed to be the rate-limiting factor in telomerase activity, ectopic expression of hTERT in cells lacking active telomerase has been shown to restore enzymatic activity, highlighting the critical role of hTERT in telomerase function [36,37]. Telomerase activation wields the potential to rescue critically short telomeres from a state of crisis, thereby extending a cell’s replicative life span, but it has also been suggested that telomerase aids in maintaining the telomere’s protective protein cap and may even signal to the cell to continue dividing, even when the telomeres are not concurrently lengthening [38]. hTERT expression and telomerase activity therefore clearly illustrate an important element of telomere dynamics. In this study, ovarian tumor tissue exhibited substantial hTERT expression and telomerase activity, while adjacent normal and benign ovarian tissues showed no hTERT mRNA or corresponding telomerase activity. This marked distinction clearly underscores the import of telomerase in cancer, although its precise role may not yet be fully understood [39,40]. Whether the enzyme is rescuing critically short telomeres from a point of crisis, maintaining telomeric integrity, or performing some other signaling function that prompts the cell to continue dividing, the presence and activation of telomerase in cancer cannot be denied. The striking difference between telomerase expression and activity in tumor tissue and their non-malignant counterparts unequivocally highlights a pivotal role telomerase appears to play in the development and progression of ovarian carcinoma. Dysfunctional telomere structure, via telomere shortening and other modes, resulting in telomere fusions, leading to genomic instability via breakage-fusion-bridge cycles, is likely a driving factor in the subsequent mis-regulation of telomerase activity in tumor cells.

In conclusion, the results presented here provide direct evidence of telomere dysfunction in human ovarian carcinoma. This data strongly suggests that telomere dysfunction occurs at a high frequently in ovarian cancer and likely plays a pivotal role in driving ovarian tumor development through breakage-fusion-bridge cycles leading to genomic instability. Deregulation of telomerase activity, likely due to this and other modes of genomic instability, are then required to maintain chromosome ends to promote malignant transformation of these damaged cells. Augmenting our knowledge regarding when and how telomere dysfunction occurs in a variety of human tissue types will likely enable us to elucidate more specific processes and common pathway in cancer development. This knowledge will be a key component in improved clinical methods of detection, treatment, and prevention of this and other diseases associated with telomere dysfunction.

## Figures and Tables

**Table 1:** Breakdown of patient samples received from IU Cancer Center Tissue Bank at the IU Simon Cancer Center. hTERT mRNA, telomerase activity, telomere length, and telomere fusion information from subsequent tests is included.

No.	Age (Yr)	Histologic Type	Tissue Location	hTERT mRNA Expression	Telomerase Activity	Telomere Length (Kb)	Telomere Fusion
N1	54	normal	Ovary	−	−	7.21	−
N2	36	normal	Ovary	−	−	7.77	+
N3	44	normal	Ovary	−	−	7.36	−
N4	52	normal	Ovary	−	−	6.12	−
N5	46	normal	Ovary	−	−	7.80	−
N6	42	normal	Ovary	−	−	8.26	−
N7	80	normal	Uterus myometrium	−	−	8.98	−
N8	58	normal	Ovary	−	−	8.83	−
N9	55	normal	Ovary	−	−	7.25	−
N10	48	normal	Endometrium	−	−	7.90	−
N11	51	normal	Endometrium	−	−	6.90	−
N12	67	normal	Ovary	−	−	8.98	−
N13	50	normal	Ovary	−	−	7.59	−
N14	47	normal	Uterus myometrium	−	−	7.21	−
B1	25	benign, fibrothecoma, Brenner’s tumor	Ovary	−	−	5.82	−
B2	26	benign	Ovary	−	−	6.26	−
B3	41	benign	Ovary	−	−	7.32	−
B4	30	benign	Ovary	−	−	7.90	−
B5	82	benign	Ovary	−	−	6.83	−
B6	65	benign	Ovary	−	−	7.20	−
B7	35	benign	Ovary	−	−	8.53	+
B8	62	benign	Ovary	−	−	7.28	−
B9	79	benign	Ovary	−	−	6.15	+
B10	69	benign	Ovary	−	−	9.23	−
B11	39	benign	Ovary	−	−	6.21	−
B12	45	benign	Ovary	−	−	4.44	−
B13	60	benign	Ovary	−	−	6.39	−
B14	51	benign	Ovary	−	−	6.35	−
B15	66	benign	Ovary	−	−	6.09	−
B16	81	benign	Ovary	−	−	6.90	−
B17	60	benign	Ovary	−	−	5.82	−
B18	48	benign	Ovary	−	−	6.26	−

**Table 2:** Ovarian tumor tissues.

No.	Age (Yr)	Histologic Type	Grade	hTERT mRNA Expression	Telomerase Activity	Telomere Length	Telomere Fusion
OT1	42	papillary serous cystadenocarcinoma, nos	II	+	+	4.80	−
OT2	75	papillary serous cystadenocarcinoma, nos	III	+	+	4.34	+
OT3	78	papillary serous cystadenocarcinoma, nos - low malignant potential	II	+	+	4.42	+
OT4	65	mixed papillary serous cystadenocarcinoma and endometriod carcinoma	III	+	+	3.89	+
OT5	50	serous cystadenocarcinoma, nos	II	+	+	3.64	+
OT6	80	serous cystadenocarcinoma, nos	III	+	+	3.79	+
OT7	52	mixed carcinoma (endometriod and mucinous), nos	I	+	+	6.50	+
OT8	56	carcinoma, nos, borderline serous tumor with intraepithelial carcinoma	III	+	+	5.21	+
OT9	80	carcinoma, nos, 80% tumor, 20% necrosis	III	+	+	5.22	+
OT10	77	carcinoma, nos	I	+	+	6.83	−
OT11	58	carcinoma, nos	high	+	−	6.16	−
OT12	65	carcinoma, nos	high	+	+	4.60	+
OT13	57	adenocarcinoma, nos	III	+	+	6.17	−
OT14	70	adenocarcinoma, nos	high-grade	+	+	7.14	−
OT15	56	adenocarcinoma, nos	II	+	+	5.45	−
OT16	46	adenocarcinoma, nos	II	+	+	7.06	−
OT17	60	adenocarcinoma, nos	IIIC	+	+	4.81	−
OT18	58	papillary adenocarcinoma, nos	I	+	+	4.47	−
OT19	79	papillary carcinoma, nos	IIIC	+	+	3.66	−
OT20	52	clear cell adenocarcinoma, nos	III	−	+	5.51	−
OT21	44	clear cell adenocarcinoma, nos	III	+	+	3.72	−
OT22	50	serous carcinoma	III	+	+	4.42	−
OT23	48	serous adevocarcinoma	III	+	+	4.30	+
OT24	56	poor differentiated adenocarcinoma	III	+	+	4.57	−
OT25	60	Papillary serous adenocarcinoma	III	+	+	6.79	−
OT26	70	Endometrioid	III	+	+	4.63	+
OT27	65	Papillary serous adenocarcinoma	III	+	+	5.27	−
OT28	64	Papillary serous carcinoma	III	+	+	5.41	−
OT29	69	mucinous	III	+	+	6.93	−
OT30	58	mixed with papillary serous carcinoma and clear cell	III	+	+	5.92	+
OT31	74	Papillary serous adenocarcinoma	III	+	+	6.60	+
OT32	60	serous cystadenocarcinoma, nos	II	+	+	4.47	+
OT33	61	poor differentiated adenocarcinoma	III	+	+	5.66	−
OT34	82	Papillary serous carcinoma	III	+	+	5.40	+
OT35	64	Papillary serous carcinoma	II	+	+	4.80	+
OT36	53	serous carcinoma	III	+	+	4.97	−
